# Genetic prediction of causal association between serum bilirubin and hematologic malignancies: a two-sample Mendelian randomized and bioinformatics study

**DOI:** 10.3389/fonc.2024.1364834

**Published:** 2024-04-08

**Authors:** Lihua Lu, Luting Luo, Xiang Li, Wanying Liu, Boheng Wu, Qing Cai, Jiazheng Li, Yan Huang, Yanxin Chen, Yongzhi Zheng, Jianda Hu

**Affiliations:** ^1^ Fujian Medical University Union Hospital, Fuzhou, Fujian, China; ^2^ The Second Affiliated Hospital of Fujian Medical University, Quanzhou, Fujian, China; ^3^ Institute of Precision Medicine, Fujian Medical University, Fuzhou, Fujian, China

**Keywords:** total bilirubin, direct bilirubin, hematologic malignancies, acute myeloid leukemia, Mendelian randomized, bioinformatics

## Abstract

**Introduction:**

An increasing number of cohort studies have shown a correlation between serum bilirubin and tumors, but no definitive causal relationship has been established between serum bilirubin and hematological malignancies.Therefore, the aim of the present study was to assess the causal relationship of serum bilirubin, including total bilirubin (TBIL) and direct bilirubin (DBIL), with hematological malignancies, including leukemia, lymphoma, and myeloma.

**Methods:**

We used a genome-wide association study (GWAS) collection of TBIL, DBIL, and hematological malignancies data. Using two-sample Mendelian randomization(MR), we assessed the impact of TBIL and DBIL on hematological malignancies. For this study, the inverse variance weighting method (IVW) was the primary method of MR analysis. In the sensitivity analysis, the weighted median method, MR Egger regression, and MR-PRESSO test were used. To understand the mechanisms behind TBIL and DBIL, we used three different approaches based on screening single nucleotide polymorphisms (SNPs) and their associated genes, followed by bioinformatics analysis.

**Results:**

The IVW test results showed evidence of effects of TBIL (odds ratio [OR]: 4.47, 95% confidence interval [CI]: 1.58-12.62) and DBIL (OR: 3.31, 95% CI: 1.08-10.18) on the risk of acute myeloid leukemia (AML).The findings from bioinformatics indicated that TBIL could potentially undergo xenobiotic metabolism through cytochrome P450 and contribute to chemical carcinogenesis.

**Discussion:**

In this study, two-sample MR analysis revealed a causal relationship between TBIL, DBIL, and AML.

## Graphical Abstract

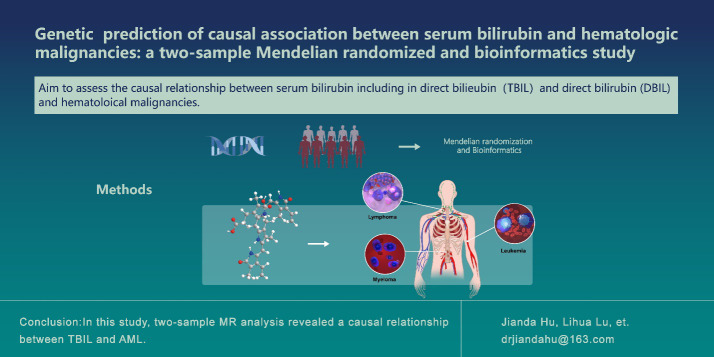

## 1 Introduction

Hematologic malignancies constitute a group of cancers, including leukemia, lymphoma, and myeloma. As a result of the onset timing and genetic profile of leukemia, the disease is classified into acute and chronic forms, further categorized into acute lymphoblastic leukemia (ALL), acute myeloid leukemia (AML), chronic lymphocytic leukemia (CLL) and chronic myeloid leukemia (CML). Lymphomas are categorized into types such as diffuse large B-cell lymphoma (DLBCL), follicular lymphoma (FL), NK/T-cell lymphoma (NKTL), and Hodgkin’s lymphoma (HL), while the category of myeloma includes multiple myeloma (MM) (1).

In economically developed countries, hematological malignancies are diagnosed as the fourth most common type of cancer among men and women (2). The progression and advancement of blood cancer depend on a combination of environmental and genetic factors (3). A wide range of genetic variants and chromosomal abnormalities contribute to hematological malignancies. For example, known myeloid leukemia susceptibility disorders include DDX41, ELANE, CEBPA, ETV6, and so on (4). Mutations in ETV6, PAX5, and TP53 increase the risk of lymphoid malignancies (5). Due to the lack of prospective data on serum bilirubin as a test, most subsequent recommendations are based on expert opinion and experience (6). Therefore, more prospective data are needed to explore the factors that influence hematological malignancies.

Serum bilirubin, produced from the breakdown of heme in aging red blood cells, is recognized as a significant endogenous antioxidant (7). In the tumor immune microenvironment, bilirubin may play a role as an important endogenous antioxidant (8). A study showed that serum bilirubin levels were negatively correlated with the risk of lung, colon, and cervical cancer (9). It has been shown that elevated bilirubin independently predicts outcomes in childhood ALL, with patients exhibiting higher bilirubin levels experiencing poorer treatment results (10). It is, however, still unclear whether serum bilirubin levels cause hematological malignancies, and thus further research is needed.

An emerging method in medicine, Mendelian randomization (MR), is being used to investigate causal relationships between environmental and medical factors (11). In MR, genotypes are established at birth, and therefore are less likely to be influenced by confounding factors. Similar to randomized controlled trials (RCTs), MR assigns genetic variants according to randomization, independent of environmental factors, assuring equal distribution of known and unknown confounders (12). In addition, genome-wide association studies (GWAS) can provide data for MR studies. Accordingly, MR studies offer benefits such as convenience, speed, large sample sizes, and significant reduction of confounding (13). In our study, we used a two-sample MR approach to assess the causal impact of TBIL and DBIL on hematological malignancies, including AML, CML, CLL, FL, HL, NKTL, and MM.

## 2 Materials and methods

### 2.1 Study design and data sources

#### 2.1.1 Study design

An MR approach examines whether a changeable risk factor and a result are causally linked, utilizing the randomness of genetic variation during conception as an observational experiment. Our study mainly included two steps. The first was to use the two-sample MR method to clarify the causal relationship between exposure and outcome. The second step was to explore the mechanisms by which exposure affects the outcome using bioinformatics methods.

In this study, MR was used to assess causal relationships of TBIL and DBIL with the risk of hematologic malignancies including AML, CLL, CML, DLBCL, FL, HL, NKTL, and MM. A valid instrumental variable, which is a single nucleotide polymorphism (SNP), must adhere to the following three principal criteria: (1) The relevance criterion for SNPs is that they need to correlate with exposure; (2) The independence criterion states that SNPs should be independent of confounding factors; (3) The exclusion restriction is that SNPs determine outcomes simply based on exposure.

To elucidate the mechanisms by which the exposures to TBIL and DBIL influence outcomes, we employed three methods to identify genes associated with these exposures. Subsequently, we applied bioinformatics techniques for Kyoto Encyclopedia of Genes and Genomes (KEGG) and Gene Ontology (GO) analyses, and to pinpoint the core genes involved.

**Figure 1:**
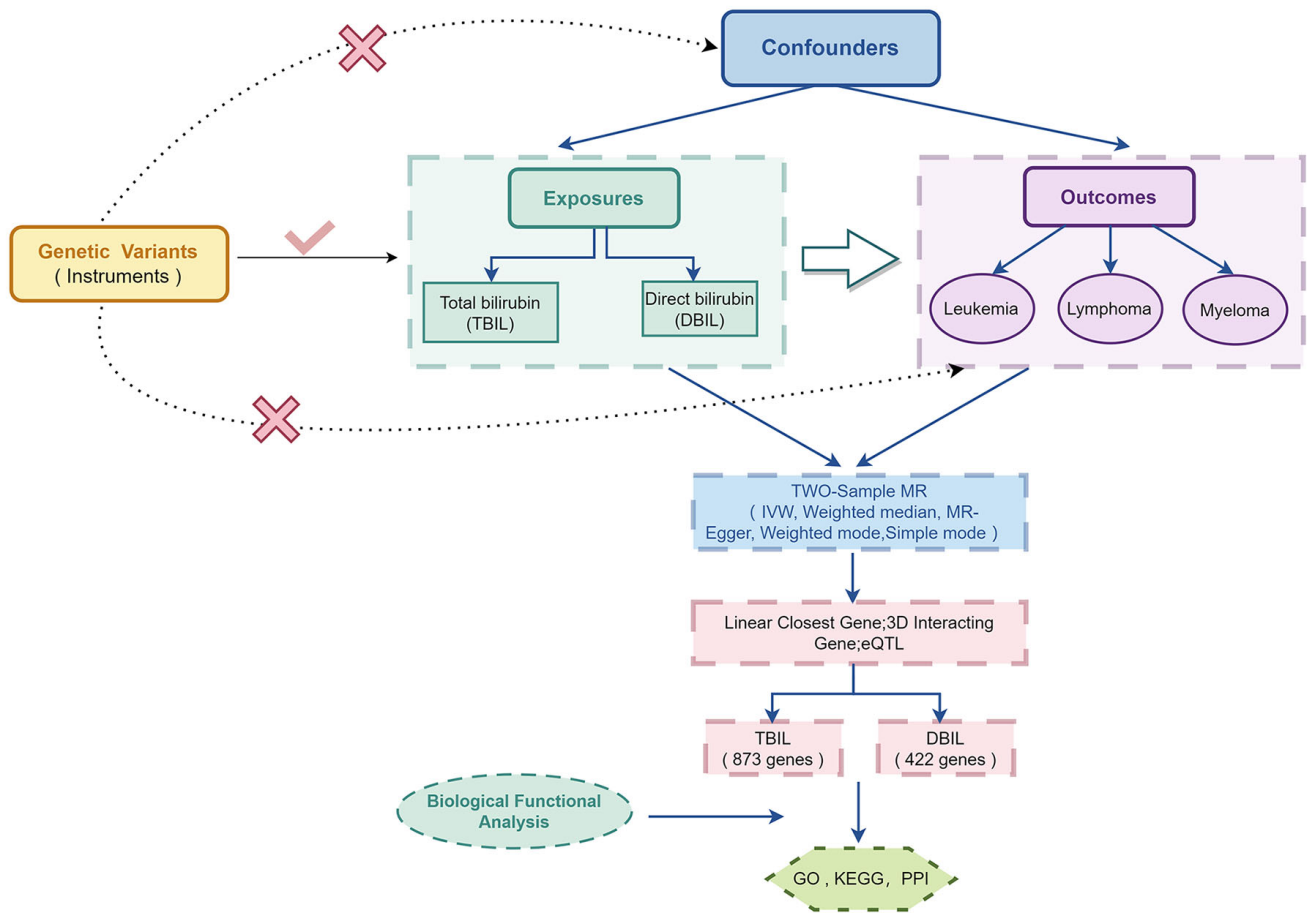
Overview of the study design in this Two-sample MR study.

The specific experimental design is shown in **Figure 1**.

#### 2.1.2 Data sources

All outcome and exposure data were from Europeans. The exposure data and the summary data for GWAS of total and direct serum bilirubin were obtained from the UK Biobank resource (available at Neale Lab UK Biobank). The GWAS for TBIL encompassed 13,585,986 SNPs. Similarly, the GWAS for DBIL comprised 13,584,679 SNPs.

Correspondingly, the outcome dataset for MM was sourced from the UK Biobank. MM dataset included 601 cases and 372,016 controls.

The outcome GWAS datasets for AML (90 cases and 218,702 controls), CLL (265 cases and 218,527 controls), CML (111 cases and 217,902 controls), DLBCL (209 cases and 218,583 controls), FL (522 cases and 218,270 controls), HL (369 cases and 218,423 controls), and NKTL (150 cases and 218,642 controls) were derived from the FinnGen dataset (available at FinnGen). The relevant data are shown in **Supplementary Table 1**.

### 2.2 Instrument selection

(1) The criterion for selecting SNPs was set at a P-value less than 5×10^-8^.
(2) For linkage disequilibrium (LD), SNPs with r^2^ < 0.01 and distances within 10,000 kb were considered.
(3) A harmonization of exposure and outcome SNPs was achieved, and SNPs related to the outcome were removed.
(4) The F statistic quantifies the strength of the instruments for each exposed SNP, obtained by dividing the square of β by the square of σ, where β represents the relationship between SNP and exposure. In general, F statistic values above 10 were considered strongly associated with exposure (14).
(5) We searched for all the SNPs in Phenoscanner (www.phenoscanner.medschl.cam.ac.uk.) (15), a database that has many results from big GWAS studies with more than 65 billion links and more than 150 million genetic changes, to investigate whether these SNPs might be associated with other traits at the genome-wide significance level (P<5x10^-8^) that could be potential confounders. We found no SNPs were clearly associated with hematologic malignancies.

### 2.3 MR analysis

In order to assess exposure and outcome causally, we used five MR methods, including the inverse-variance weighted method, MR Egger method, weighted median method, weighted mode method, and simple mode method (16–19). To perform MR analysis in this study, inverse variance weighting (IVW) was used as the main method. The IVW approach assumes that all the chosen instrumental variables are valid, indicating that they satisfy the three key assumptions of MR.

In order to verify the robustness of the causal association, a range of sensitivity analyses were conducted. The tests included Cochran’s Q test and I^2^ statistics for heterogeneity testing (considering a P< 0.05 or an I^2^ > 50% significant), MR PRESSO, pleiotropy test, and leave-one-out test. The MR-PRESSO program detects horizontal pleiotropy outliers in MR summary data. As a result of this method, horizontal pleiotropy can be not only identified but also removed. Further, it enables comparison of the results of MR analysis before and after corrections (20). The credibility of the findings is enhanced by the leave-one-out test, which shows that the results are not excessively reliant on any individual genetic variation (21). As a result, we used the leave-one-out test to rule out the influences of single-variant SNPs.

All statistical analyses were conducted using R software (version 4.2.2) and two packages (TwoSampleMR and MR-PRESSO).

### 2.4 Bioinformatics analysis

Three distinct methodologies were employed—linear closest gene, three-dimensional interacting gene, and eQTL—on the 3DSNP online platform (available at https://omic.tech/3dsnpv2/) to analyze SNPs that are closely associated with TBIL (137 SNPs) and DBIL (84 SNPs) exposure. This comprehensive approach enabled us to identify 837 genes with a close association with TBIL and 422 genes associated with DBIL.

Subsequently, function enrichment analyses were conducted, including the KEGG pathway and GO enrichment analyses. Additionally, we constructed a protein-protein interaction network using the String online database and identified the top 10 core genes using Cytoscape’s Hub plugin.

## 3 Results

### 3.1 Two−sample MR analysis of serum bilirubin and the risk of hematologic malignancies

To investigate the effect of serum bilirubin on the risk of hematologic malignancies, we conducted an MR analysis of TBIL and DBIL on the risk of AML, CLL, CML, DLBCL, FL, HL, NKTL, and MM.

After the screening of instrumental variables, we identified 137 SNPs associated with TBIL and 84 SNPs associated with DBIL, respectively (P < 5e-8). Subsequently, using F-statistics, sensitivity analyses, leave-one-out test, and so on, we screened the SNPs that could be used to analyze the exposure and outcome as follows: (1) Exposure to TBIL: AML (125 SNPs), CLL (125 SNPs). CML (126 SNPs), DLBCL (126 SNPs), FL (126 SNPs), HL (125 SNPs), NKTL (125 SNPs), and MM (111SNPs); (2) Exposure to DBIL: AML (76 SNPs), CLL (76 SNPs), CML (75 SNPs), DLBCL (77 SNPs), FL (76 SNPs), HL (75 SNPs), NKTL (76 SNPs), and MM (67 SNPs). The relevant data are shown in **Tables 1**, **2**.

**Table 1:** MR results of TBIL (except IVW) and results of pleiotropy,heterogenity and MR-PRESSO analyses.

Exposure	Outcome		No. of SNP	Method	Effect Estimate		Pleiotropy		Heterogeneity		MR-PRESSO
**Total bilirubin**	** **				95% CI	p-value	Intercept	p-value	Q	p-value	p-value
	**Leukemia**	AML	125	MR Egger	3.47 ( 0.80 to 15.02 )	0.0982	0.0121	0.631	129.853	0.234	0.276
				Weighted median	1.99 ( 0.36 to 10.97 )	0.4256					
				Weighted mode	3.38 ( 0.65 to 17.55 )	0.1505					
				Simple mode	20.93 ( 0.74 to 592.09)	0.0770					
		CLL	125	MR Egger	1.18 ( 0.52 to 2.66 )	0.6975	0.0081	0.566	97.045	0.930	0.939
				Weighted median	0.94 ( 0.35 to 2.50 )	0.9082					
				Weighted mode	0.73 ( 0.25 to 2.18 )	0.5760					
				Simple mode	0.93 (0.14 to 6.30 )	0.9450					
		CML	126	MR Egger	0.79 ( 0.53 to 1.09 )	0.1360	0.0153	0.359	104.581	0.841	0.895
				Weighted median	0.80 ( 0.56 to 1.59 )	0.2326					
				Weighted mode	0.81 ( 0.56 to 1.18 )	0.2752					
				Simple mode	0.48 ( 0.04 to 5.32 )	0.5517					
	**Lymphoma**	DLBCL	126	MR Egger	0.83 ( 0.64 to 1.08 )	0.1588	0.0140	0.250	104.869	0.836	0.894
				Weighted median	0.87 ( 0.66 to 1.14 )	0.2990					
				Weighted mode	0.84 ( 0.64 to 1.09 )	0.1913					
				Simple mode	0.71 ( 0.11 to 4.56 )	0.7223					
		FL	126	MR Egger	1.12 ( 0.95 to 1.33 )	0.1777	-0.0025	0.743	100.970	0.896	0.94
				Weighted median	1.12 ( 0.95 to 1.33 )	0.1870					
				Weighted mode	1.15 ( 0.98 to 1.36 )	0.0970					
				Simple mode	1.15 ( 0.39 to 3.40 )	0.8003					
		HL	125	MR Egger	1.23 ( 0.61 to 2.47 )	0.5625	0.0048	0.691	104.176	0.832	0.872
				Weighted median	1.11 ( 0.51 to 2.42 )	0.7991					
				Weighted mode	1.82 ( 0.57 to 2.46 )	0.6551					
				Simple mode	0.99 ( 0.24 to 4.00 )	0.9873					
		NKTL	125	MR Egger	1.23 ( 0.41 to 3.64 )	0.7131	-0.0091	0.6277	109.127	0.731	0.769
				Weighted median	1.13 ( 0.31 to 4.17 )	0.8539					
				Weighted mode	1.60 ( 0.44 to 5.85 )	0.4782					
				Simple mode	1.60 ( 0.14 to 18.87 )	0.7093					
	**Multiple myeloma**	Multiple myeloma	111	MR Egger	1.00 ( 1.00 to 1.00 )	0.9736	6.28E-06	0.6929	105.806	0.432	0.436
				Weighted median	1.00 ( 1.00 to 1.00 )	0.8656					
				Weighted mode	1.00 ( 1.00 to 1.00 )	0.8399					
				Simple mode	1.00 ( 1.00 to 1.00 )	0.7739					

**Table 2:** MR results of DBIL (except IVW) and results of pleiotropy,heterogenity and MR-PRESSO analyses.

Exposure	Outcome		No. of SNP	Method	Effect Estimate		Pleiotropy		Heterogeneity		MR-PRESSO
**Direct bilirubin**	** **				95% CI	p-value	Intercept	p-value	Q	p-value	p-value
	**Leukemia**	AML	76	MR Egger	2.07 ( 0.44 to 9.76 )	0.3602	0.03014023	0.391	71.438	0.397	0.449
				Weighted median	2.50 ( 0.45 to 13.70 )	0.2908					
				Weighted mode	2.68 ( 0.55 to 13.07 )	0.2280					
				Simple mode	9.07 ( 0.30 to 277.85 )	0.2106					
		CLL	76	MR Egger	1.96 ( 0.81 to 4.77 )	0.1410	-0.011736642	0.560	54.226	0.904	0.885
				Weighted median	3.06 ( 1.13 to 8.26 )	0.0273					
				Weighted mode	3.41 ( 1.20 to 9.72 )	0.0245					
				Simple mode	1.69 ( 0.27 to 10.64 )	0.5769					
		CML	75	MR Egger	2.47 ( 0.54 to 11.29 )	0.2460	-0.028605128	0.377	65.439	0.566	0.601
				Weighted median	2.52 ( 0.42 to 14.93 )	0.3093					
				Weighted mode	2.37 ( 0.45 to 12.39 )	0.3090					
				Simple mode	2.37 ( 0.12 to 46.08 )	0.5699					
	**Lymphoma**	DLBCL	77	MR Egger	0.84 ( 0.60 to 1.18 )	0.3192	0.002705949	0.888	80.583	0.182	0.361
				Weighted median	0.84 ( 0.63 to 1.14 )	0.2646					
				Weighted mode	0.83 ( 0.60 to 1.13 )	0.2411					
				Simple mode	1.15 ( 0.18 to 7.29 )	0.8810					
		FL	76	MR Egger	1.06 ( 0.56 to 2.00 )	0.8500	0.012582724	0.383	59.369	0.789	0.84
				Weighted median	1.16 ( 0.60 to 2.24 )	0.6559					
				Weighted mode	1.28 ( 0.71 to 2.30 )	0.4196					
				Simple mode	1.21 ( 0.36 to 4.01 )	0.7521					
		HL	75	MR Egger	1.25 ( 0.52 to 3.01 )	0.6187	-0.010125208	0.571	59.264	0.766	0.794
				Weighted median	0.85 ( 0.32 to 2.27 )	0.7500					
				Weighted mode	0.88 ( 0.31 to 2.50 )	0.8030					
				Simple mode	0.51 ( 0.09 to 3.00 )	0.4552					
		NKTL	76	MR Egger	0.79 ( 0.24 to 2.56 )	0.6905	-0.012366491	0.644	50.444	0.955	0.968
				Weighted median	0.60 ( 0.16 to 2.24 )	0.4468					
				Weighted mode	0.70 ( 0.20 to 2.46 )	0.5843					
				Simple mode	3.23 ( 0.25 to 42.01 )	0.3742					
	**Multiple myeloma**	Multiple myeloma	67	MR Egger	1.00 ( 1.00 to 1.00 )	0.8340	-5.16E-06	0.821	55.728	0.597	0.624
				Weighted median	1.00 ( 1.00 to 1.00 )	0.5980					
				Weighted mode	1.00 ( 1.00 to 1.00 )	0.8272					
				Simple mode	1.00 ( 1.00 to 1.00 )	0.5905					

In order to determine the precision and dependability of our MR analysis, we conducted tests for pleiotropy and heterogeneity, verifying that the selected SNPs in our study were devoid of both pleiotropy and heterogeneity. Concurrently, we employed the MR-PRESSO tool to identify and rectify any aberrant SNPs that could introduce bias. Notably, the P-values of MR-PRESSO in this study all exceeded the threshold of 0.05, underscoring the trustworthiness of our findings (**Tables 1**, **2**).

**Figure 2:**
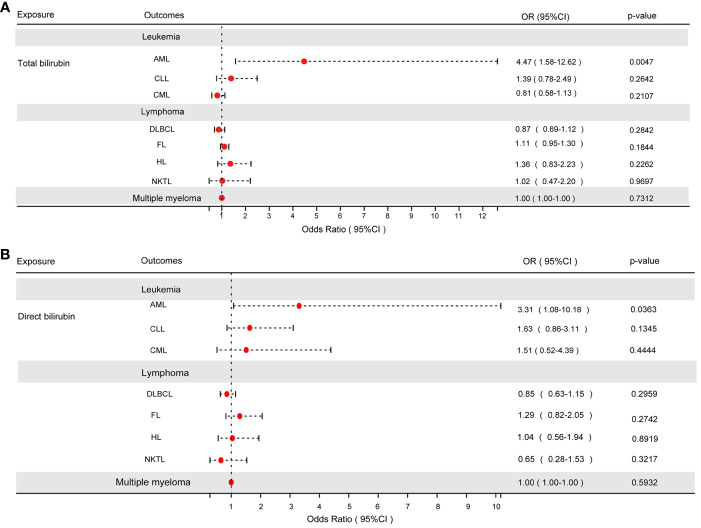
**(A)** The results of the IVW analysis of between TBIL and hematologic malignancies. **(B)** The results of the IVW analysis of between DBIL and hematologic malignancies.

When the P-value fell below 5×10^-8^, the IVW test revealed a substantial link between TBIL and AML (odds ratio [OR]: 4.47, 95% confidence interval [CI]: 1.58-12.62, P = 4.68e-3), as depicted in **Figure 2A**. However, TBIL showed no significant causal connection with other diseases listed in **Table 1**. In a similar vein, for DBIL, statistical significance in relation to AML (OR: 3.31, 95% CI: 1.08-10.18, P = 3.63e-2) under the same P-value threshold, as demonstrated by the IVW test in **Figure 2B**. Similarly, DBIL did not exhibit a significant causal relationship with additional diseases mentioned in **Table 2**.

### 3.2 Biological functional analysis

**Figure 3:**
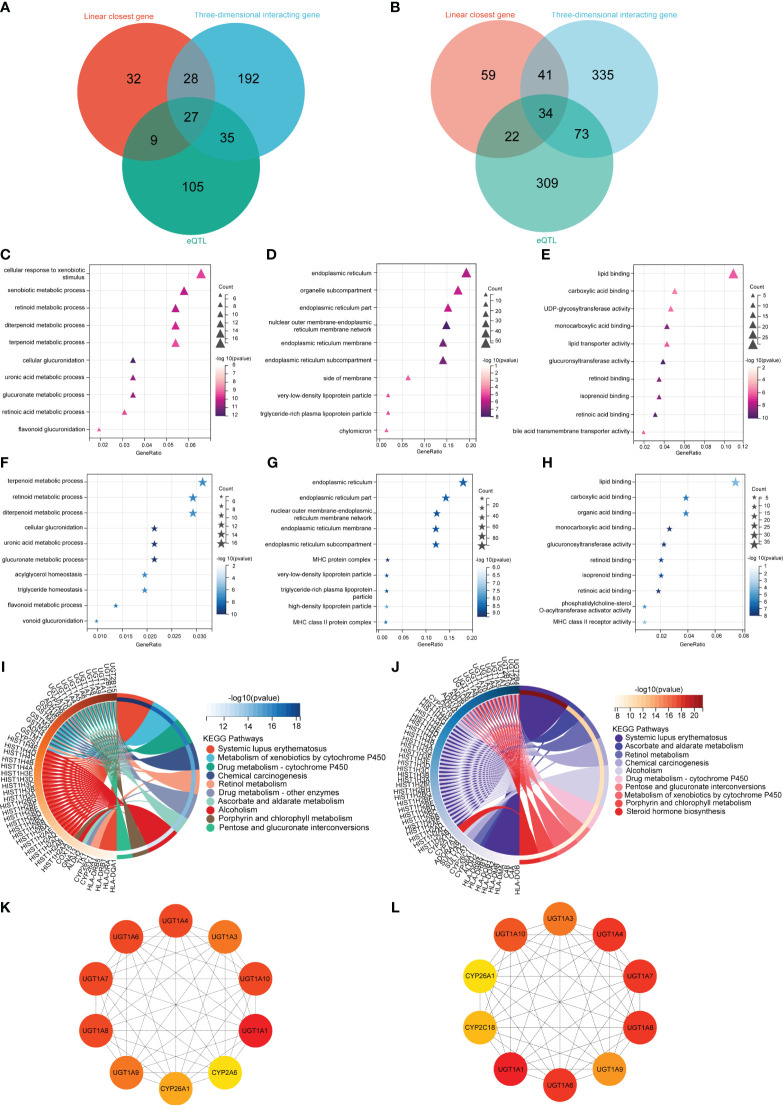
Biological functional analysis. The DBIL **(A)** and TBIL **(B)** venn diagram illustrates three methodologies employed for the identification of genes linked to DBIL and TBIL. The GO enrichment analysis of DBIL encompassed three main categories: **(C)** Biological Process, **(D)** Cell Component, and **(E)** Molecular Function. The GO enrichment analysis of TBIL encompassed three main categories: **(F)** Biological Process, **(G)** Cell Component, and **(H)** Molecular Function. The KEGG enrichment analysis of DBIL **(I)** and TBIL **(J)**. A protein-protein interaction network to identify the top 10 fundamental genes associated with DBIL **(K)** and TBIL **(L)**.

Using three different gene mapping strategies, the 3DSNP online platform (https://omic.tech/3dsnpv2/) identified genes related to 137 SNPs linked to TBIL in the MR study. The identification of TBIL-related genes included the following: (1) According to the linear closest gene mapping (genes within 2 kb upstream or downstream of the SNP), 156 genes were identified. (2) Using the three-dimensional interaction gene (genes interacting with the SNP via 3D chromatin loops), a total of 483 genes were identified. (3) The eQTL-based mapping implicated 438 genes. After the removal of duplicate genes, the three methods identified 873 genes associated with TBIL, of which 34 genes were identified simultaneously by all three methods (**Figure 3A**). The identification of DBIL-related genes included the following: (1) According to the linear closest gene mapping, 96 genes were identified. (2) Using the three-dimensional interaction gene, 282 genes were identified. (3) The eQTL-based mapping implicated 176 genes. After the removal of duplicate genes, the three methods identified 873 genes associated with TBIL, of which 34 genes were identified simultaneously by all three methods, and 422 genes associated with DBIL, of which 27 genes were identified simultaneously by all three methods (**Figure 3B**).

In order to investigate the possible mechanism leading to AML, all 873 genes associated with TBIL identified by the methods above were analyzed using GO and KEGG at Sangerbox 3.0, an open online site (22). To further understand the functions of these genes, they were classified into three categories: biological process (BP), cellular component (CC), and molecular function (MF). Additionally, signaling pathways of the genes were identified by KEGG pathway analysis.

In terms of BP, TBIL results showed the genes mainly corresponded to terpenoid metabolism, retinoid metabolism, and diterpenoid metabolism (**Figure 3F**). As for CC, TBIL results indicated that the genes primarily corresponded to the endoplasmic reticulum, endoplasmic reticulum part, nuclear outer membrane-endoplasmic reticulum membrane network, and so on (**Figure 3G**). As for MF, TBIL results showed the genes mainly mediated lipid binding, carboxylic acid binding, and organic acid binding (**Figure 3H**). In KEGG enrichment pathway analysis for TBIL, the genes were significantly enriched in systemic lupus erythematosus, ascorbate and aldarate metabolism, retinol metabolism, chemical carcinogenesis, and so on (**Figure 3J**).

As for BP, DBIL results showed the genes mainly occurred during cellular response to xenobiotic stimulus, xenobiotic metabolism, and retinoid metabolism (**Figure 3C**). For CC, DBIL data indicated that the genes mainly corresponded to the endoplasmic reticulum, organelle parts, and endoplasmic reticulum parts (**Figure 3D**). MF revealed DBIL-related genes mainly involved in lipid binding, carboxylic acid binding, and UDP-glycosyltransferase activity (**Figure 3E**). According to the KEGG enrichment pathway analysis for DBIL, the genes were significantly enriched in systemic lupus erythematosus, xenobiotic metabolism-cytochrome P450, and drug metabolism-cytochrome P450, and so on (**Figure 3I**).

Using Cytoscape’s Hub plugin, the top 10 core genes for TBIL were UGT1A1, UGT1A4, UGT1A6, UGT1A3, UGT1A7, UGT1A8, UGT1A9, UGT1A10, CYP2C18, and CYP26A1 (**Figure 3L**). Similarly, the top 10 core genes for DBIL were UGT1A1, UGT1A3, UGT1A4, UGT1A6, UGT1A7, UGT1A8, UGT1A9, UGT1A10, CYP2A1, and CYP26A1 (**Figure 3K**).

## 4 Discussion

In our research, we explored the involvement of TBIL and DBIL in the development of hematological malignancies using a two-sample MR approach. We found that TBIL and DBIL were causally related to AML. Further, we found that both TBIL and DBIL were risk factors for AML, and the probability of developing AML increased with increases in TBIL.

Hereditary elevations of bilirubin can cause unconjugated hyperbilirubinemia, including Crigler-Najjar syndrome (23, 24) and Gilbert syndrome (25), as well as conjugated hyperbilirubinemia, including Dubin-Johnson syndrome (26) and Rotor syndrome (27). It turns out that both Crigler-Najjar and Gilbert syndromes are caused by a lack of glucuronosyltransferase (UGT1A1), which is responsible for the conversion of bilirubin to bilirubin diglycosidic acid, making bilirubin less soluble in water (23–25). Researchers have found that UGT1A1 genotype plays a role in the clinical outcomes of cytarabine-treated intermediate-risk AML patients. A research has demonstrated that UGT1A1 mutations in pediatric leukemia can result in the onset of unconjugated hyperbilirubinemia, potentially exacerbating the condition (28). Additionally, research has indicated that in childhood leukemia, the allele frequency of UGT1A1*6 was notably elevated in the hyperbilirubinemia cohort compared to the non-hyperbilirubinemia cohort, thereby heightening the likelihood of adverse outcomes (28). Díaz-Santa et al. found that AML patients carrying the UGT1A1*28 purity variant, linked to reduced UGT1A1 activity, exhibited lower overall survival rates (29). Unexpectedly, the findings of Díaz-Santa and colleagues contradict those reported by Chen and team. In a comparative trial conducted by Chen P et al., it was observed that carriers of the UGT1A1*6 and UGT1A1*28 polymorphisms exhibited significantly higher rates of complete remission in the context of cytarabine treatment for AML among 726 adult patients. Specifically, the complete remission rates for carriers of UGT1A1*6 and UGT1A1*28 were 66.9% and 68.5%, respectively, in contrast to normal subjects. Furthermore, carriers of either UGT1A1*6 or UGT1A1*28 alleles were associated with a significantly reduced risk of non-complete remission (P= 1.7 *10^-4^) and improved overall survival (P =0.040) (30). The variations in results could be attributed to the distinct populations under investigation, as Chen et al. concentrated on Asian populations while Díaz-Santa et al. focused on European populations. This study similarly examines European populations and aligns with the conclusions drawn by Díaz-Santa et al. Additionally, researchers have found that AML cases have a significant increase in UGT1A4 (31).

In Dubin-Johnson syndrome, the ABCC2 gene, which encodes ATP-binding cassette subfamily member C, is altered. According to Loscocco et al., the ABCC2 rs3740066 genotype is associated with a swifter molecular response level 3 (MR3) in CML patients taking nilotinib (32). In the study of Martino et al., how transporter gene polymorphisms, including ABCC2, affect the risk of MM was investigated (33). The Rotor syndrome is caused by bi-allelic mutations in the SLCO1B1 and SLCO1B3 genes. These mutations impair the functioning of the organic anion-transporting polypeptides 1B1 (OATP1B1) and 1B3 (OATP1B3) located on the sinusoidal membranes of liver cells, disrupting the uptake of bilirubin by these cells (34). A research study in Poland revealed that MM patients possessing the GG genotype of the SLCO1B1 gene’s A388G SNP showed prolonged survival when treated with melphalan-prednisone, compared to other therapies. However, this genetic variant does not elevate the risk of developing MM (35).

We conducted pathway enrichment analysis on genes associated with TBIL and DBIL, which involved gene clustering methodologies. We explored potential biological mechanisms linking DBIL and TBIL to AML.

Based on analyses for DBIL, we found that the BP were related to cellular responses to xenobiotic stimuli, with a significant involvement of the KEGG pathway in the metabolism of xenobiotics by cytochrome P450. According to a review, the cytochrome P450 enzyme family is responsible for converting xenobiotics into polar substances, which increases the risk of AML. The study stresses the importance of CYP2E1, a cytochrome P450 member, in the progression of AML, suggesting its potential as a therapeutic and diagnostic target (36). Some studies have explored the fact that CYP2E1 gene variants can indirectly affect bilirubin metabolism. For instance, a study examined how CYP2E1 gene polymorphisms affected bilirubin levels by influencing liver function (37).Additionally, we found that the KEGG pathway was enriched for chemical carcinogenesis. Researchers have discovered that chemical carcinogens, such as formaldehyde and benzene, contribute to the progression of hematological malignancies, thereby increasing the risk of developing malignant hematological diseases (38, 39).

The analysis of GO enrichment for TBIL indicated significant associations between BP and the terpenoid metabolic process, retinoid metabolic process, diterpenoid metabolic process and cellular glucronidation. Based on analyses for TBIL, we found that in common with DBIL, the KEGG pathway was enriched for the metabolism of xenobiotics by cytochrome P450 and chemical carcinogenesis. Additionally, we also found that KEGG pathway was enriching in pentose and glucuronate interconversions. A study of porphyrin metabolism in ALL, non-Hodgkin’s lymphoma (NHL), and Hodgkin’s lymphoma (HL) showed a significant increase in the activity of 5-aminolevulinic acid dehydratase (ALAD) and Urinary porphyrinogen synthetase (URO-S), increased concentrations of total porphyrins, protoporphyrin, and urinary protoporphyrin in the blood, as well as a decrease in hemoglobin levels in patients with ALL, as compared with healthy controls (40).

In this study, we found that TBIL and DBIL were important risk factors for AML. In the screening of core genes, TBIL and DBIL showed little difference and KEGG pathway enrichment overlapped with each other. In order to enhance the validity of our findings, we employed three methodologies to screen for genes linked to TBIL and DBIL and conducted GO enrichment and KEGG pathway enrichment analyses to investigate underlying mechanisms. The common mechanism of action of TBIL and DBIL may be the metabolism of xenobiotics by cytochrome P450 and chemical carcinogenesis. The strength of this study is that large-scale GWAS of TBIL, DBIL, and AML were used, which provides suitable statistical power. In addition, the studies included in the MR analyses mostly included individuals of European origin, which reduces population stratification bias. Furthermore, the method is less susceptible to confounding since genetic information is used as an instrumental variable.

MR studies, however, may have limited validity due to pleiotropy. Our findings remain stable across a variety of MR approaches, without any signs of directional pleiotropy. However, this study has certain limitations, including (1) For the reverse two-sample MR, we followed a strict P-value threshold of less than 5×10^-8^ to ensure accuracy. However, this strict criterion led to insufficient SNPs for the analysis. As a result, we identified a relatively small number of SNPs linked to exposure. Therefore, we did not investigate the impact of hematological malignancies on TBIL and DBIL levels. (2) Due to its limited size and origin from a non-European population, indirect bilirubin, GWAS data are not addressed in this study. (3) Due to the lack of ALL data available, the causal relationship between TBIL and DBIL was not discussed in this study.

For a complete understanding of the generalizability of our study findings, we should consider several factors. Firstly, the susceptibility to diseases, including acute myeloid leukemia (AML), can vary among different populations due to variations in genetic background, lifestyle, and environmental exposures. As a result, our results may need to be interpreted cautiously when applied to populations with distinct characteristics from our own. Secondly, the temporal aspect should also be taken into account. For instance, long-term exposure to certain risk factors may have different effects on AML risk compared to short-term exposure. This highlights the importance of considering the duration of exposure in the assessment of disease risk. Finally, it is crucial to consider the impact of varying levels of exposure. It is thought that varying levels of bilirubin and exposure to chemicals may affect the risk of AML in different ways. The relationship between exposure levels and disease risk should be carefully considered when applying our findings to varying scenarios.

## 5 Conclusion

According to our MR analysis, TBIL and DBIL were causally associated with the risk of AML. The mechanisms underlying the cancer were possibly associated with cytochrome P450’s metabolism of xenobiotics and pathways leading to chemical carcinogenesis.

In terms of clinical implications, these findings could be used to develop screening methods for individuals with elevated blood bilirubin levels, potentially allowing early detection and treatment of AML.

## Data availability statement

The original contributions presented in the study are included in the article/**Supplementary Material**. Further inquiries can be directed to the corresponding authors.

## Author contributions

LHL: Conceptualization, Data curation, Formal analysis, Investigation, Methodology, Software, Supervision, Writing – original draft, Writing – review & editing. LTL: Data curation, Investigation, Methodology, Software, Supervision, Writing – review & editing, Writing – original draft. XL: Writing – review & editing, Writing – original draft, Conceptualization, Data curation, Investigation, Methodology, Software, Supervision. WL: Data curation, Methodology, Supervision, Writing – original draft. BW: Data curation, Methodology, Software, Writing – original draft. QC: Data curation, Investigation, Methodology, Software, Writing – original draft. JL: Investigation, Software, Validation, Writing – original draft. YH: Data curation, Methodology, Project administration, Supervision, Writing – original draft. YC: Formal analysis, Project administration, Supervision, Validation, Writing – original draft. YZ: Formal analysis, Funding acquisition, Resources, Supervision, Validation, Visualization, Writing – review & editing. JH: Formal analysis, Funding acquisition, Project administration, Resources, Supervision, Validation, Visualization, Writing – review & editing.

## Supplementary material

Supplementary Figure 1 Funnel plot of DBIL associated with hematological malignancies.

Supplementary Figure 2 Forest plot of DBIL associated with hematological malignancies.

Supplementary Figure 3 Leave-one-out plot of DBIL associated with hematological malignancies.

Supplementary Figure 4 Scatter plot of DBIL associated with hematological malignancies.

Supplementary Figure 5 Funnel plot of TBIL associated with hematological malignancies.

Supplementary Figure 6 Forest plot of TBIL associated with hematological malignancies.

Supplementary Figure 7 Leave-one-out plot of TBIL associated with hematological malignancies.

Supplementary Figure 8 Scatter plot of TBIL associated with hematological malignancies.
