# Supplementary figures and images for: Genetic prediction of causal association between serum bilirubin and hematologic malignancies: a two-sample Mendelian randomized and bioinformatics study
Source: Front Oncol. 2024 Apr 8;14:1364834. doi: 10.3389/fonc.2024.1364834 (PMC11033852; doi:10.3389/fonc.2024.1364834)

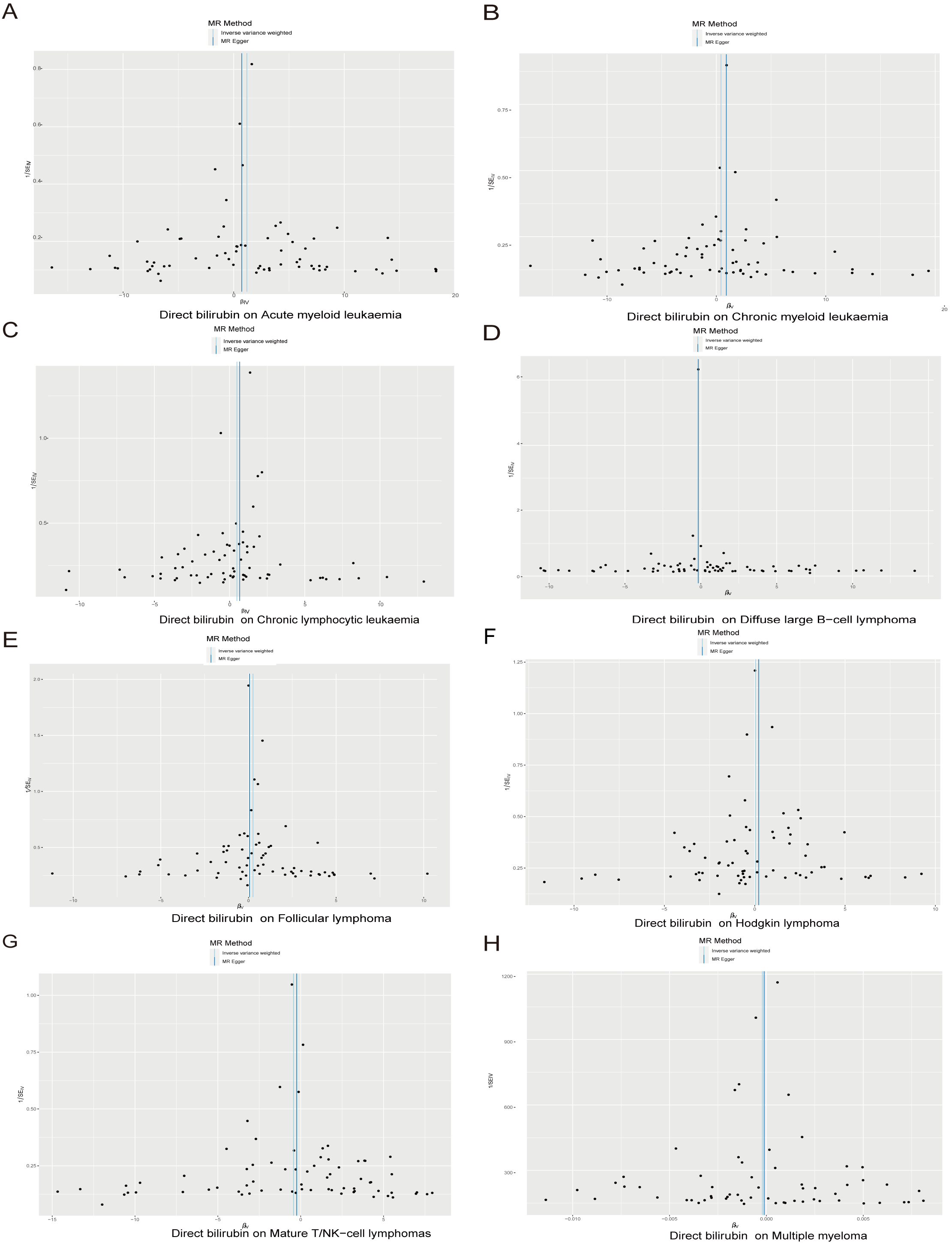

Supplement: Supplementary Figure 1 — Funnel plot of DBIL associated with hematological malignancies. [file Image_1.tif]

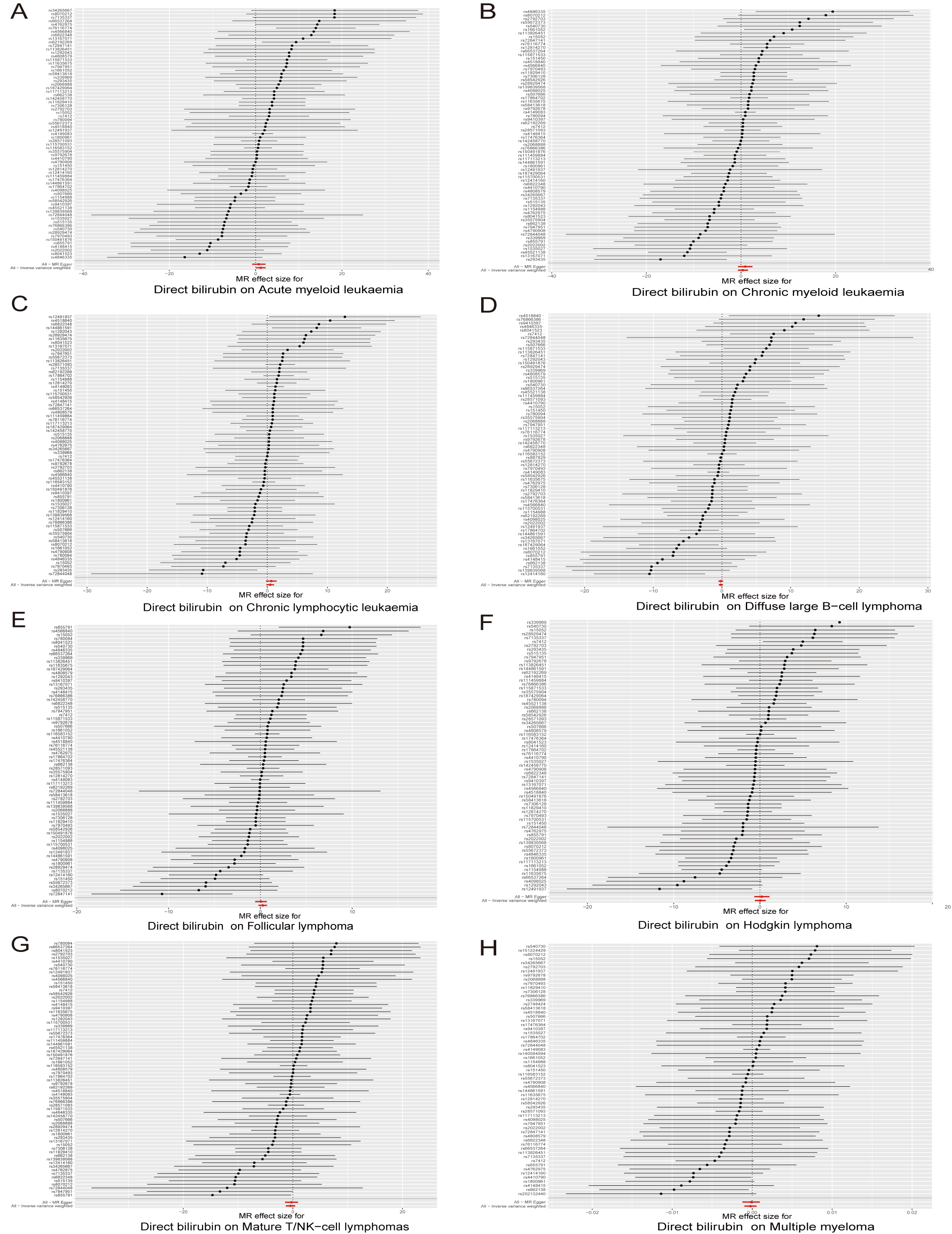

Supplement: Supplementary Figure 2 — Forest plot of DBIL associated with hematological malignancies. [file Image_2.tif]

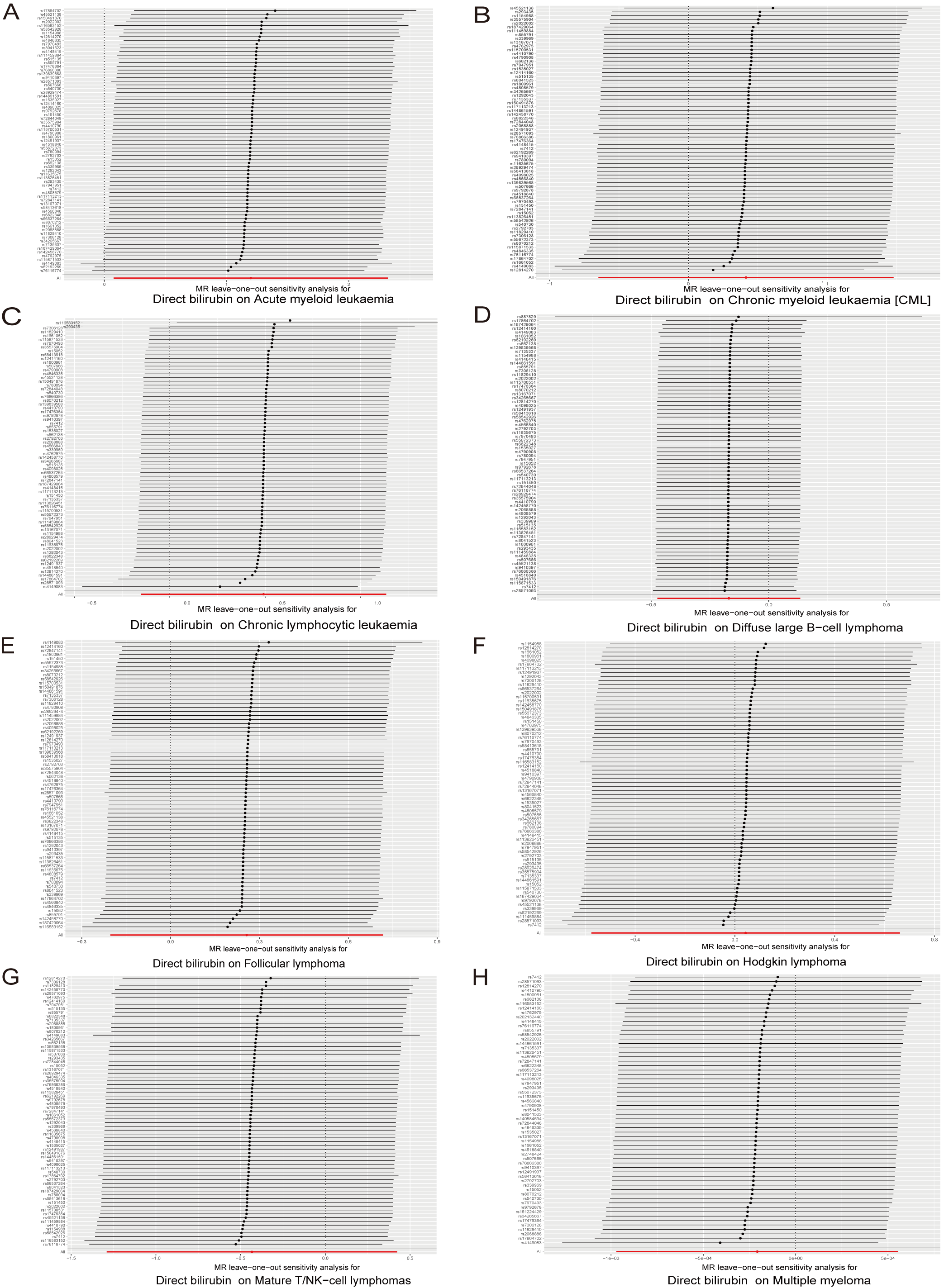

Supplement: Supplementary Figure 3 — Leave-one-out plot of DBIL associated with hematological malignancies. [file Image_3.tif]

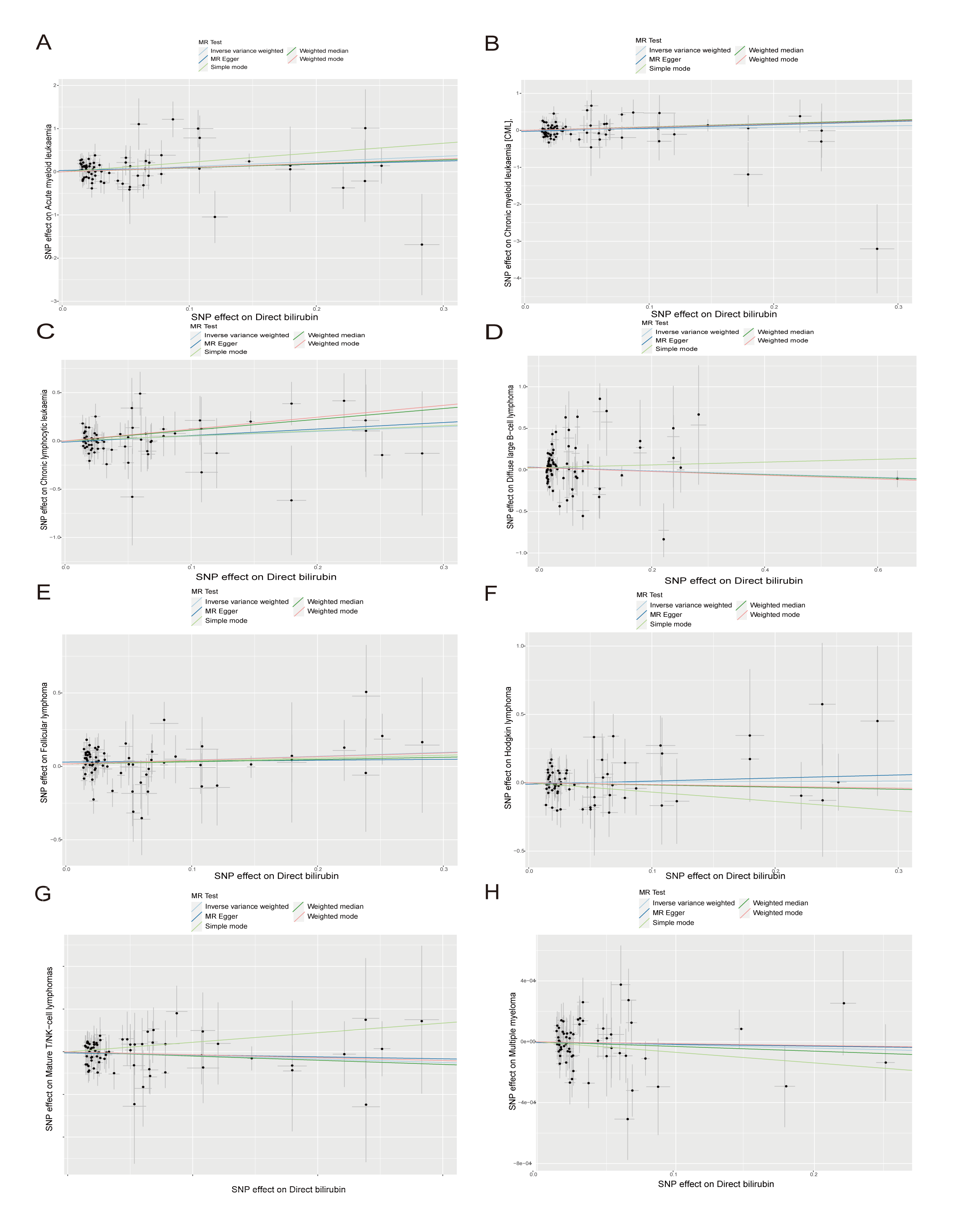

Supplement: Supplementary Figure 4 — Scatter plot of DBIL associated with hematological malignancies. [file Image_4.tif]

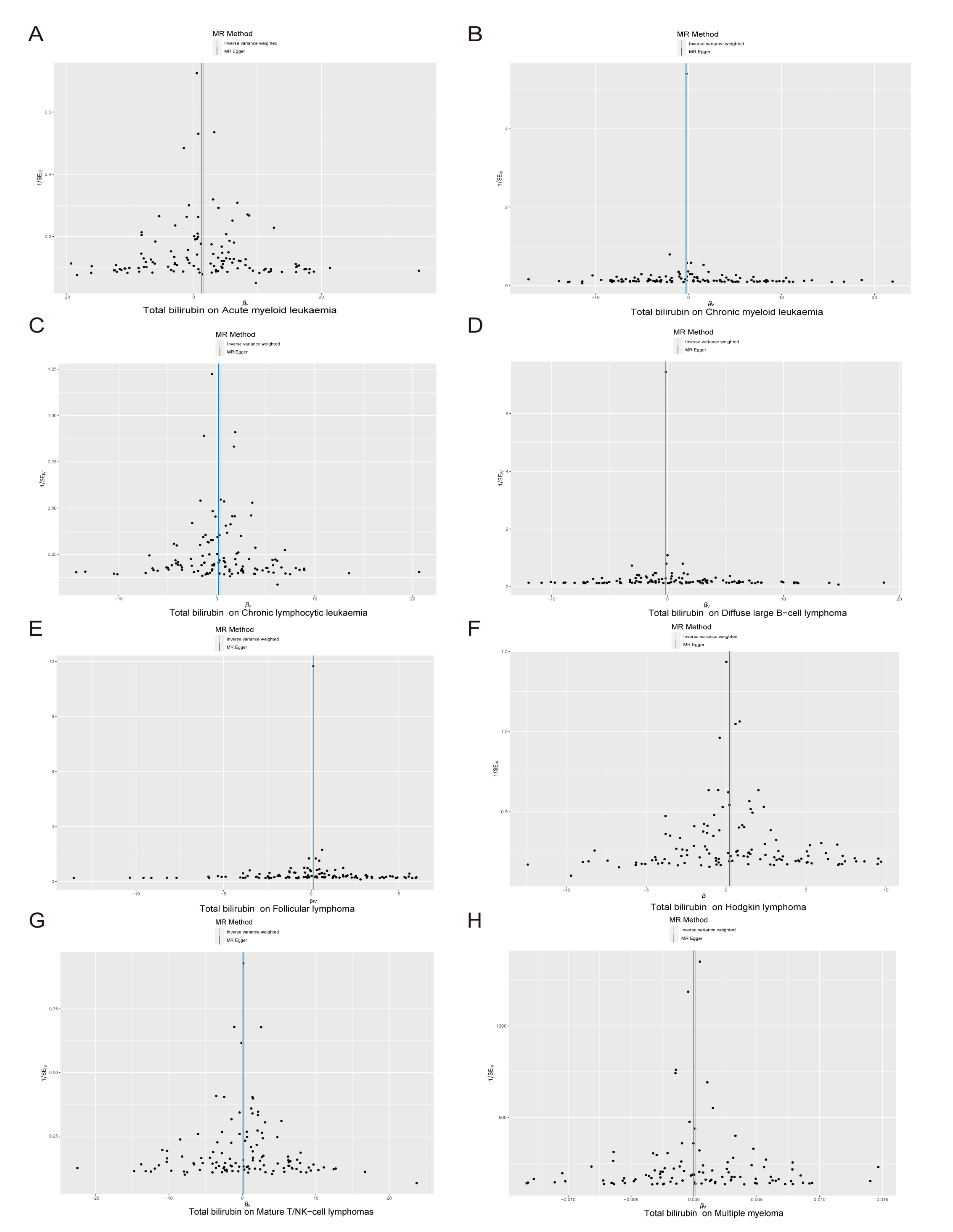

Supplement: Supplementary Figure 5 — Funnel plot of TBIL associated with hematological malignancies. [file Image_5.tif]

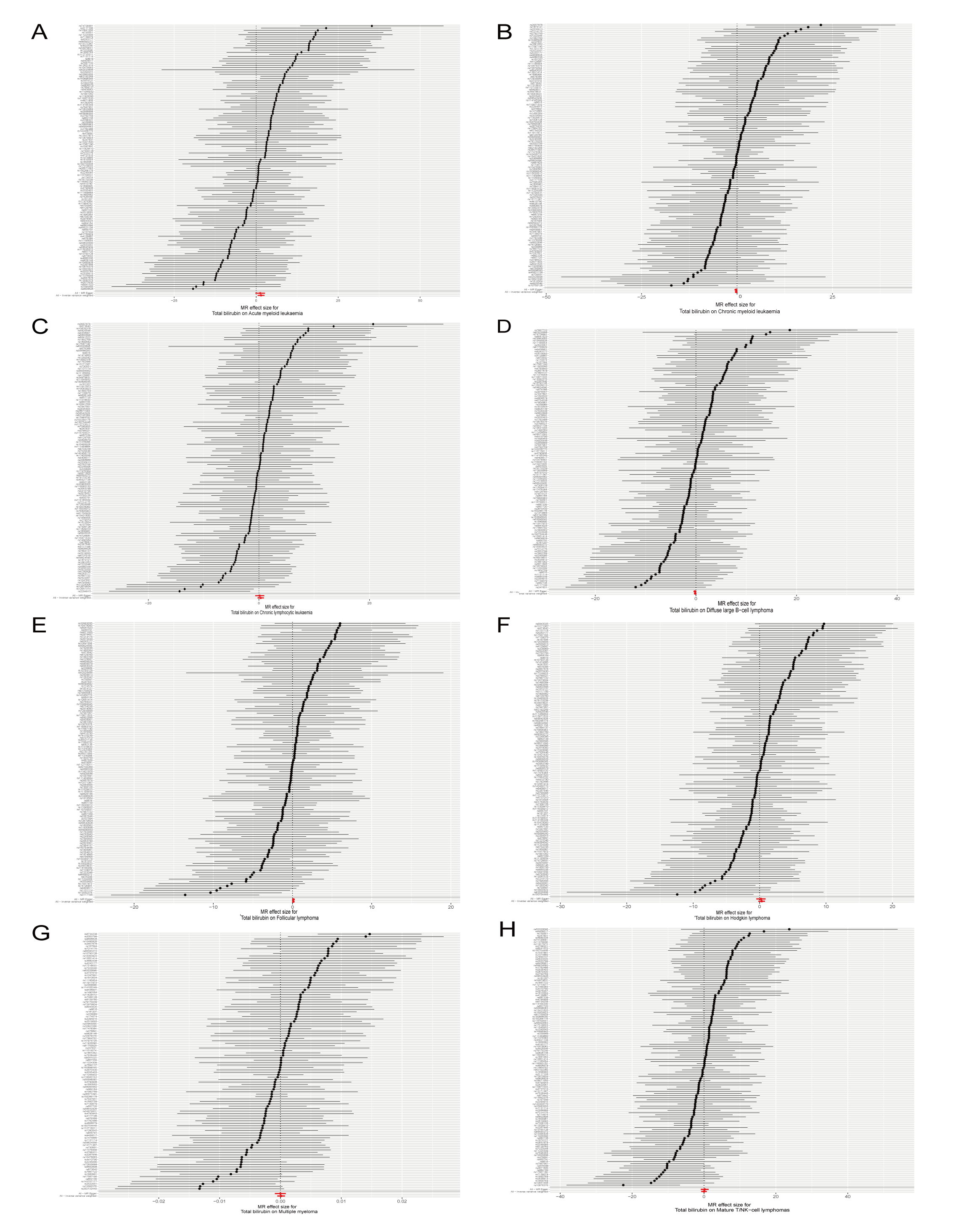

Supplement: Supplementary Figure 6 — Forest plot of TBIL associated with hematological malignancies. [file Image_6.tif]

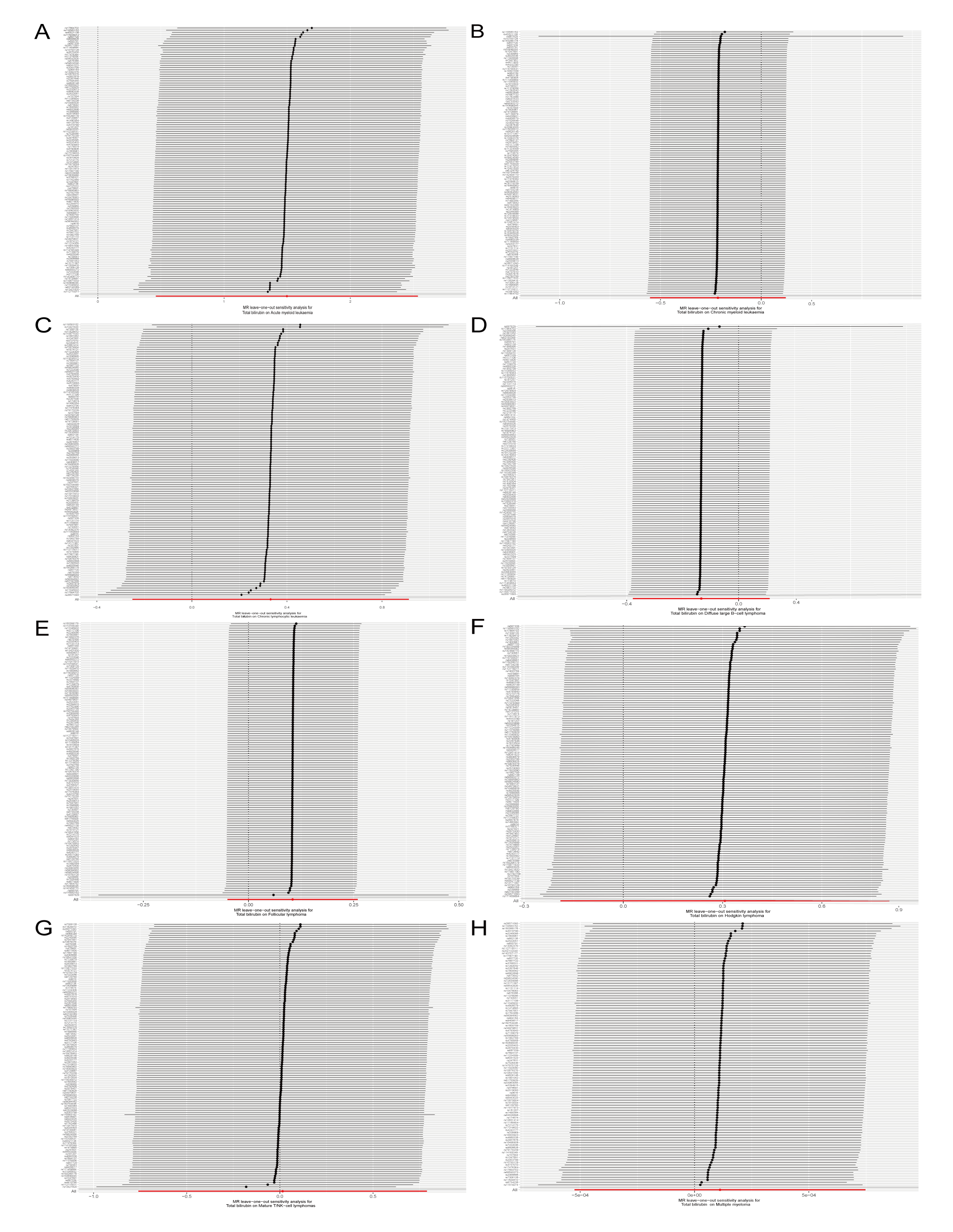

Supplement: Supplementary Figure 7 — Leave-one-out plot of TBIL associated with hematological malignancies. [file Image_7.tif]

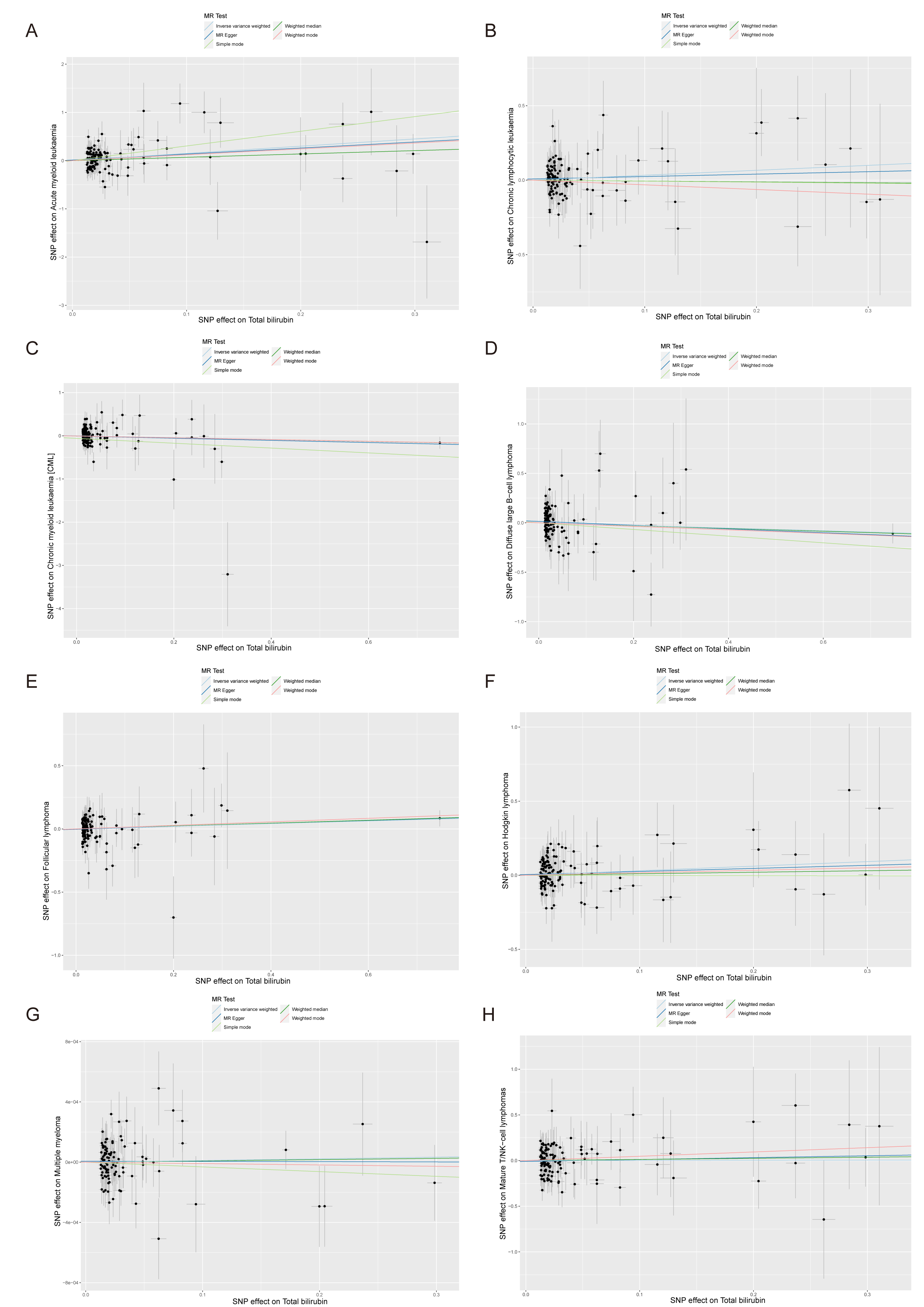

Supplement: Supplementary Figure 8 — Scatter plot of TBIL associated with hematological malignancies. [file Image_8.tif]
